# Supplementary material for: Midkine expression by stem-like tumor cells drives persistence to mTOR inhibition and an immune-suppressive microenvironment
Source: Nat Commun. 2022 Aug 26;13:5018. doi: 10.1038/s41467-022-32673-7 (PMC9418323; doi:10.1038/s41467-022-32673-7)
Supplement: Supplementary file 1 — Supplementary Information [file 41467_2022_32673_MOESM1_ESM.pdf]

AML ID	mutation
AML1098	TSC1 c.1798C>T p.Q600* at 79%
AML1162	TSC2 c2974 C>T (p.Q992*), exon 26 – in 41.33% of 1196 reads; TSC2 c5226 indels (), exon 40 – in 12.86% of 916 reads
AML1166	TSC2 c.4537G>T (p.E1513*), exon 35 - in 35% of 343 reads; TSC2 c.4442dup (p.S1482Efs*42), exon 34 - in 36% of 297 reads
AML1167	TSC2 c.1372C>T (p.R458*), exon 13 – in 47.56% of 1214 reads
AML1171	TSC2 c.1111_1119+1delinsTC (), exon 11 - in 19% of 146 reads
AML1172	TSC1 c.2074C>T (p.R692*), exon 17 - in 12% of 316 reads; TSC1 c.989_1011dup (p.I338*), exon 10 - in 4% of 304 reads

Supplementary table. Mutations identified in AML tumors. Genetic mutations identified in each AML tumor are listed.

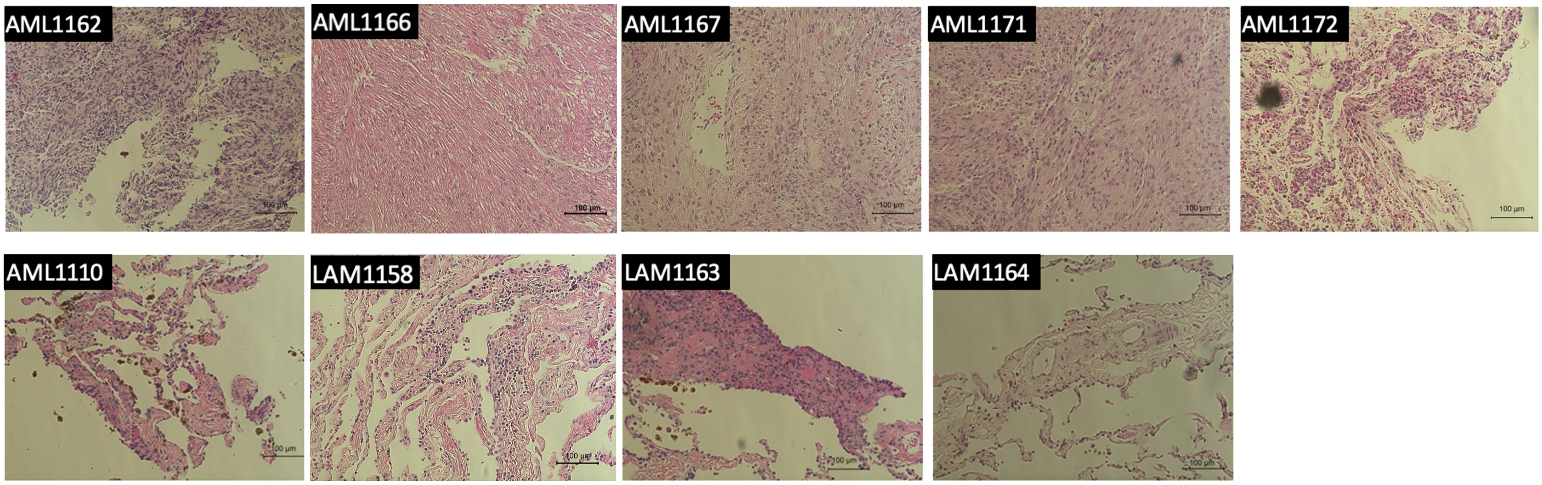

Supplementary Fig.1 Pathological images for AML and LAM specimen.
Pathological images of AML samples (upper panel) and LAM samples (lower panel). scale bar: 100μm. Each image is representative of 10 areas of each sample.

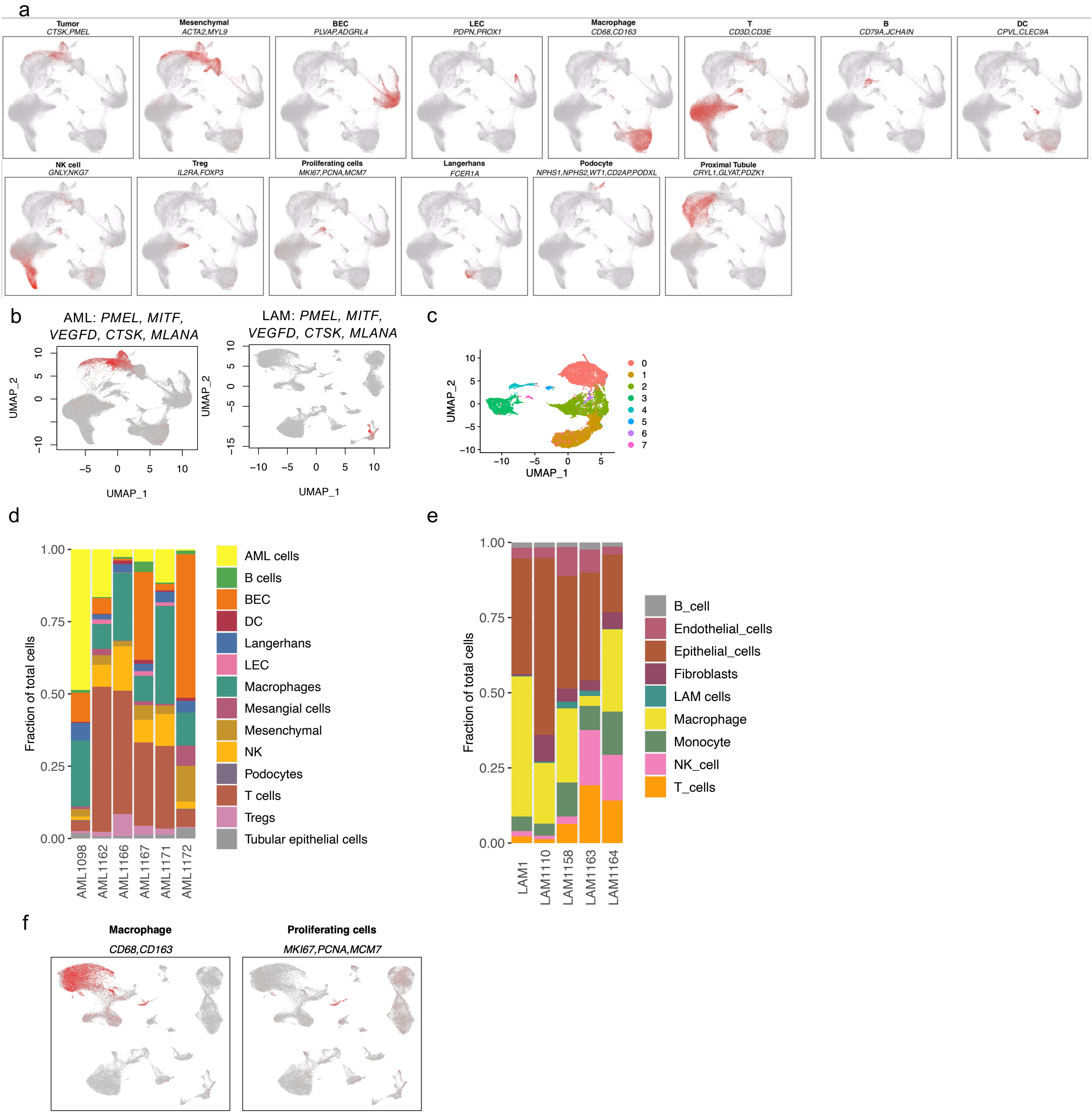

Supplementary Fig.2 Global single cell analysis of AML and LAM, related to Fig. 1.

a. Expression of representative marker genes for cell types defined in AML. Red color shows the averaged normalized expression levels of marker genes.
b. Average expression of five tumor marker genes in AML (left) and LAM (right).
c. Graph-based clustering of mesenchymal cell population results in eight clusters.
d. Cell fraction for each AML patient across cell types.
e. Cell fraction for each LAM patient across cell types.
f. Expression of representative marker genes for macrophages and proliferating cells defined in LAM. Red color shows the averaged normalized expression levels of marker genes.

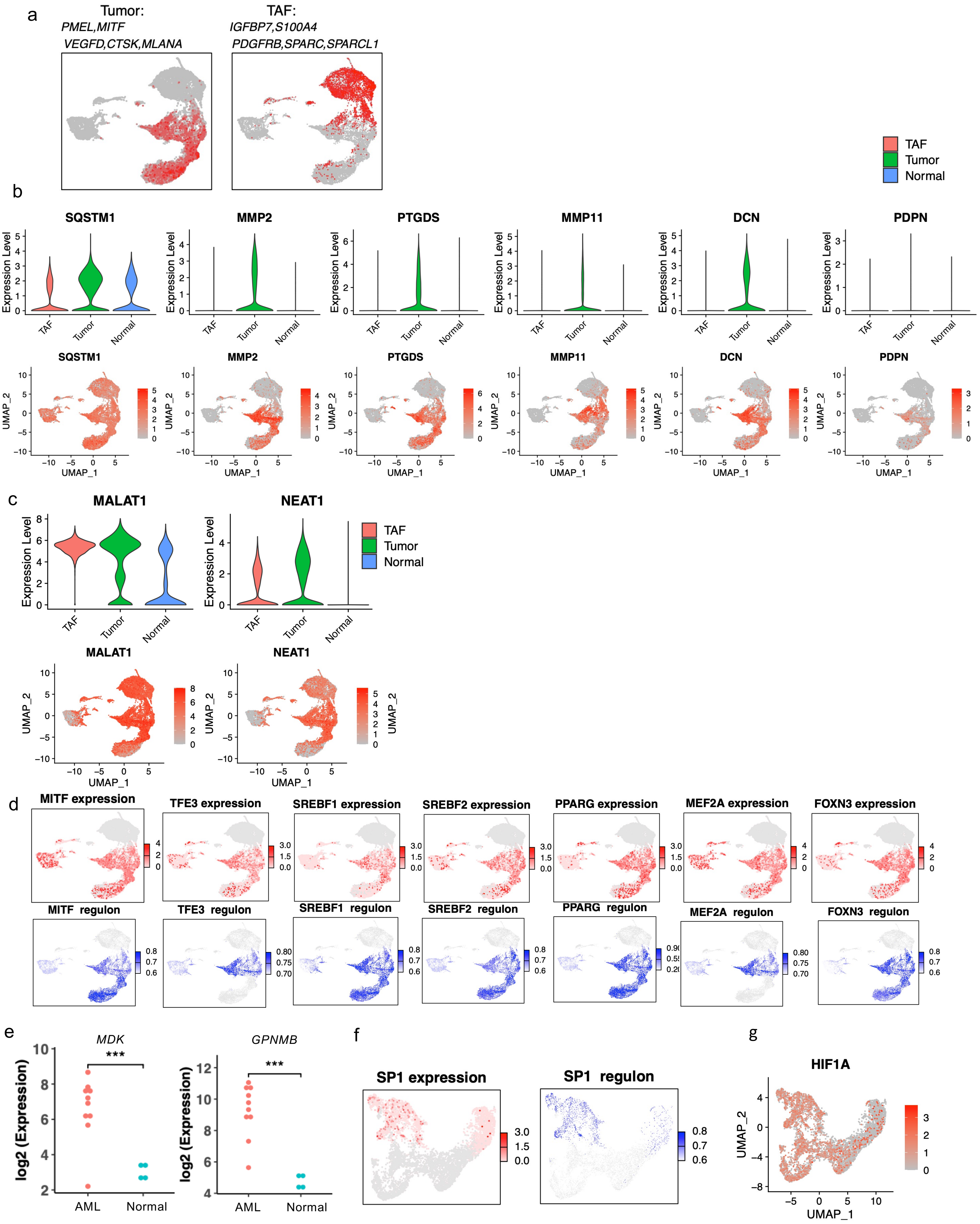

a
Tumor:
PMEL,MITF
VEGFD,CTSK,MLANA
TAF:
IGFBP7,S100A4
PDGFRB,SPARC,SPARCL1
TAF
Tumor
Normal
b
SQSTM1
MMP2
PTGDS
MMP11
DCN
PDPN
Expression Level
UMAP_1
UMAP_2
c
MALAT1
NEAT1
d
MITF expression
TFE3 expression
SREBF1 expression
SREBF2 expression
PPARG expression
MEF2A expression
FOXN3 expression
MITF regulon
TFE3 regulon
SREBF1 regulon
SREBF2 regulon
PPARG regulon
MEF2A regulon
FOXN3 regulon
e
MDK
GPNMB
log2 (Expression)
***
AML
Normal
f
SP1 expression
SP1 regulon
g
HIF1A

Supplementary Fig.3 Marker gene expression in AML, related to Fig. 1.

a. Identification of tumor cells (left) and tumor associated fibroblasts (TAF) (right) using marker genes shown. Cells expressed at least 2 marker genes above median value of corresponding genes across all cells were annotated as tumor cell or TAF, shown in red.

b. Upper panel: violin plots showing representative upregulated genes in AML cells. The y axis represents the normalized gene expression. Lower panel: feature plots of these genes.

c. Upper panel: violin plots showing expression of four long non-coding RNAs (lncRNAs). The y axis represents normalized gene expression. Lower panel: feature plots of these genes.

d. Representative regulons enriched in AML cells. First row: expression of transcription factors. Second row: regulon activities of these transcription factors.

e. Expression of *MDK* and *GPNMB* assessed by bulk RNA-Seq comparing AML tumors with normal kidneys. ***: MDK p-value=0.0001; GPNMB p-value=7.5e-06, two-sided t-test.

f. Expression and regulon activity of *SP1* in AML cells.

g. Expression of *HIF1A* in AML cells.

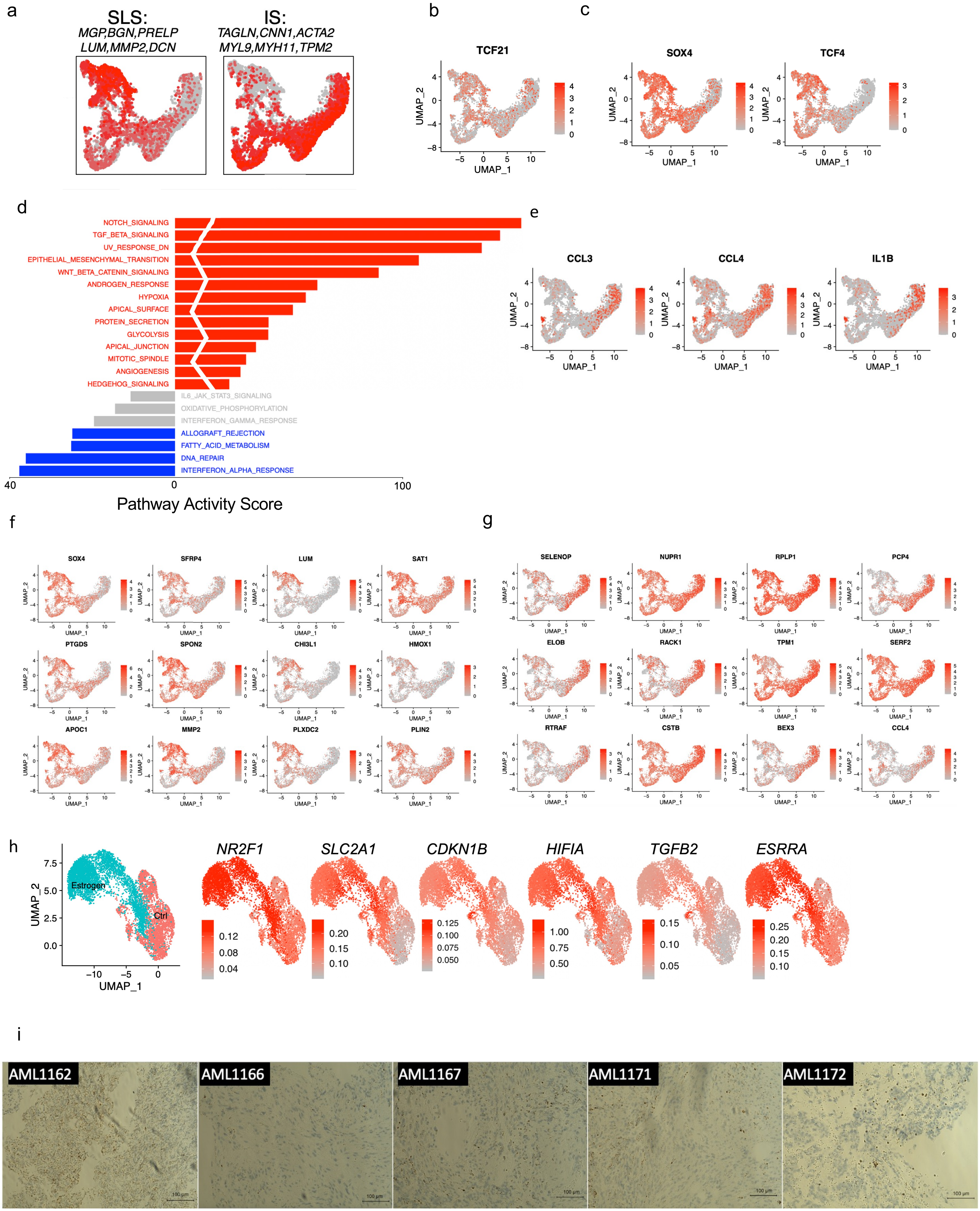
a
SLS:
MGP,BGN,PRELP
LUM,MMP2,DCN
IS:
TAGLN,CNN1,ACTA2
MYL9,MYH11,TPM2
b
TCF21
c
SOX4
TCF4
d
NOTCH_SIGNALING
TGF_BETA_SIGNALING
UV_RESPONSE_DN
EPITHELIAL_MESENCHYMAL_TRANSITION
WNT_BETA_CATENIN_SIGNALING
ANDROGEN_RESPONSE
HYPOXIA
APICAL_SURFACE
PROTEIN_SECRETION
GLYCOLYSIS
APICAL_JUNCTION
MITOTIC_SPINDLE
ANGIOGENESIS
HEDGEHOG_SIGNALING
IL6_JAK_STAT3_SIGNALING
OXIDATIVE_PHOSPHORYLATION
INTERFERON_GAMMA_RESPONSE
ALLOGRAFT_REJECTION
FATTY_ACID_METABOLISM
DNA_REPAIR
INTERFERON_ALPHA_RESPONSE
Pathway Activity Score
e
CCL3
CCL4
IL1B
f
SOX4
SFRP4
LUM
SAT1
PTGDS
SPON2
CHI3L1
HMOX1
APOC1
MMP2
PLXDC2
PLIN2
g
SELENOP
NUPR1
RPLP1
PCP4
ELOB
RACK1
TPM1
SERF2
RTRAF
CSTB
BEX3
CCL4
h
Estrogen
Ctrl
NR2F1
SLC2A1
CDKN1B
HIFIA
TGFB2
ESRRA
i
AML1162
AML1166
AML1167
AML1171
AML1172

Supplementary Fig.4 Identification of two distinct cell states in AML/LAM cells, related to Fig. 2.

a. Feature plot showing averaged expression of *MGP*, *BGN*, *PRELP*, *LUM, MMP2, DCN* (left) and *TAGLN*, *CNN1*, *ACTA2*, *MYL9*, *MYH11*, *TPM2* (right).
b. Feature plot showing expression of *TCF21*.
c. Feature plot showing expression of *SOX4* and *TCF4*.
d. Pathways enriched in SLS population, calculated using differentially expressed genes in cluster 1 (SLS) compared to cluster 2 (IS). Red: pathways enriched in SLS population; Blue: pathways enriched in IS population; x-axis: pathway activity score.
e. Feature plots showing representative inflammatory genes upregulated in the IS population.
f. Feature plots showing representative genes upregulated in the SLS population.
g. Feature plots showing representative genes upregulated in the IS population.
h. Expression of dormancy marker genes in TSC2-null 621-101 cells before and after estradiol treatment.
i. Representative RNAScope images of MDK expression in each AML tumor analyzed.

a

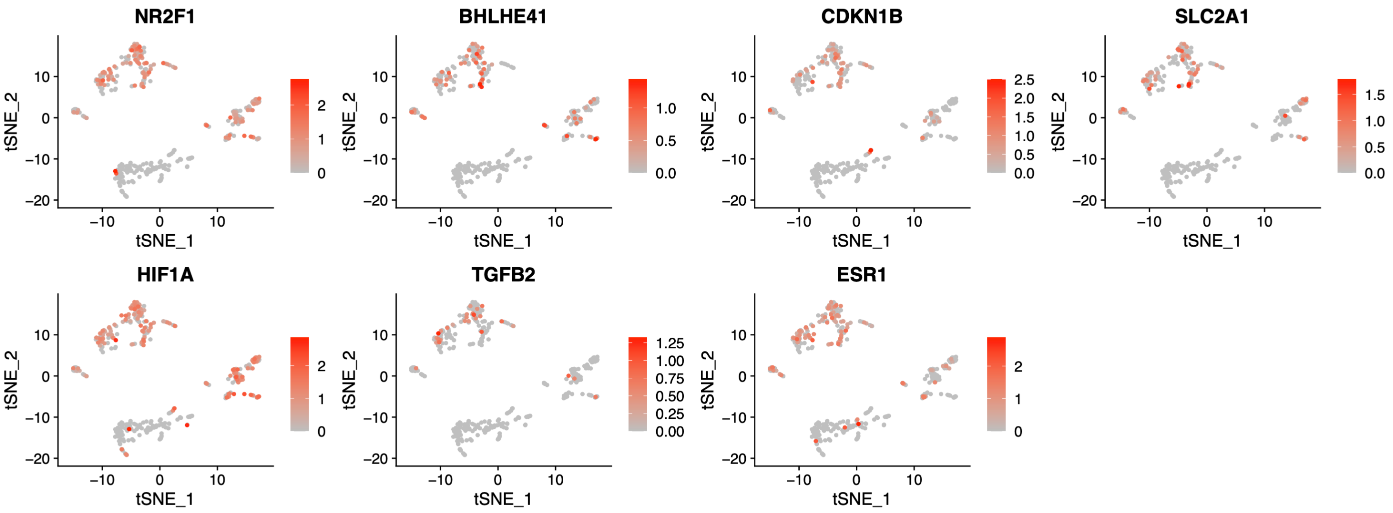

b

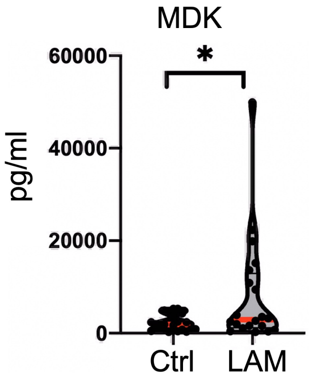

Supplementary Fig.5 Expression of marker genes in LAM, related to Fig. 2.

a. Feature plots showing expression of dormancy genes in LAM cells.

b. ELISA assessment of serum MDK levels in healthy cohorts (n=19) and in LAM patients (n=20). *: p-value=0.0361, two-sided t-test.

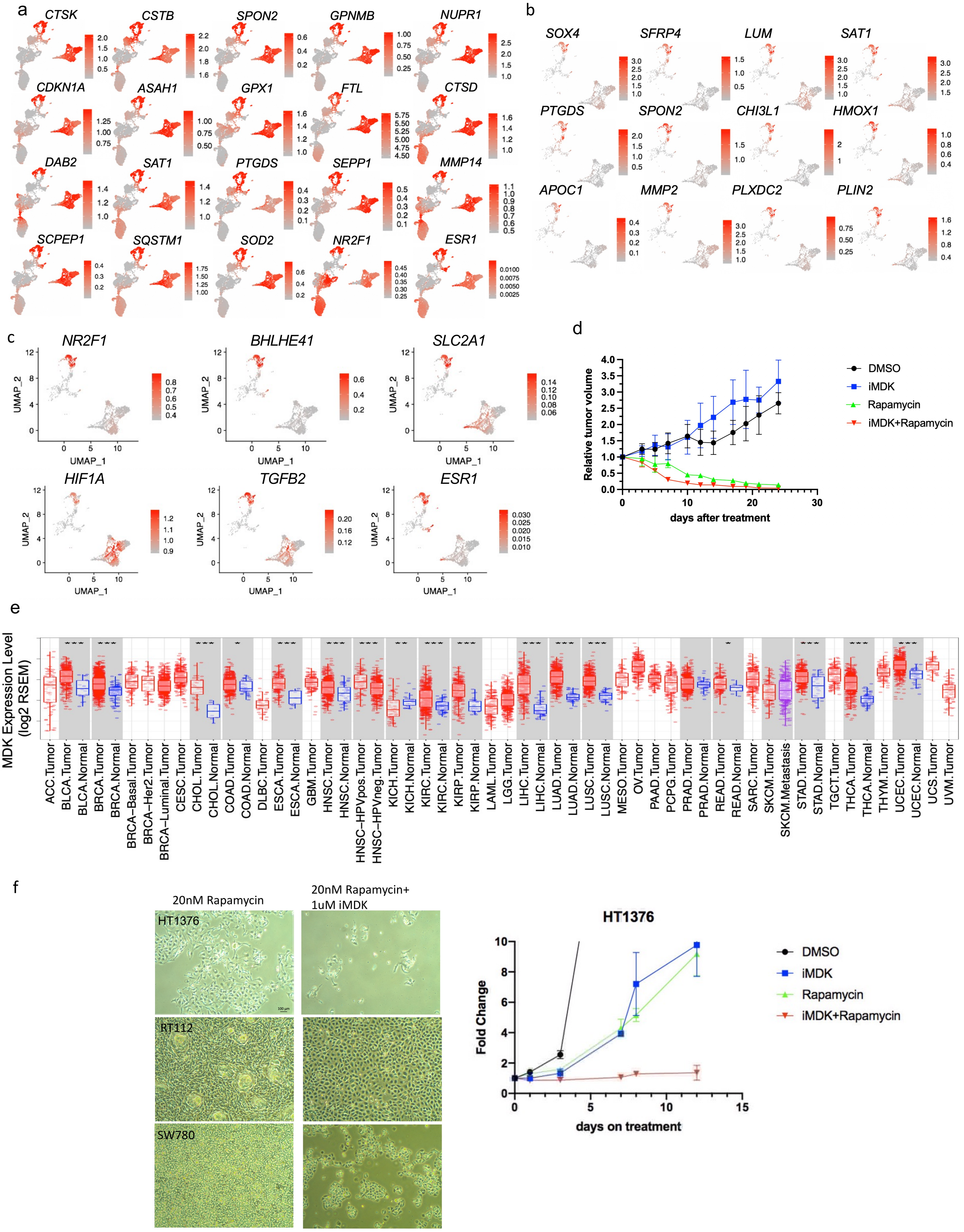
a
CTSK
CSTB
SPON2
GPNMB
NUPR1
CDKN1A
ASAH1
GPX1
FTL
CTSD
DAB2
SAT1
PTGDS
SEPP1
MMP14
SCPEP1
SQSTM1
SOD2
NR2F1
ESR1
b
SOX4
SFRP4
LUM
SAT1
PTGDS
SPON2
CHI3L1
HMOX1
APOC1
MMP2
PLXDC2
PLIN2
c
NR2F1
BHLHE41
SLC2A1
HIF1A
TGFB2
ESR1
UMAP_1
UMAP_2
d
Relative tumor volume
days after treatment
DMSO
iMDK
Rapamycin
iMDK+Rapamycin
e
MDK Expression Level (log2 RSEM)
ACC.Tumor
BLCA.Tumor
BLCA.Normal
BRCA.Tumor
BRCA.Normal
BRCA−Basal.Tumor
BRCA−Her2.Tumor
BRCA−Luminal.Tumor
CESC.Tumor
CHOL.Tumor
CHOL.Normal
COAD.Tumor
COAD.Normal
DLBC.Tumor
ESCA.Tumor
ESCA.Normal
GBM.Tumor
HNSC.Tumor
HNSC.Normal
HNSC−HPVpos.Tumor
HNSC−HPVneg.Tumor
KICH.Tumor
KICH.Normal
KIRC.Tumor
KIRC.Normal
KIRP.Tumor
KIRP.Normal
LAML.Tumor
LGG.Tumor
LIHC.Tumor
LIHC.Normal
LUAD.Tumor
LUAD.Normal
LUSC.Tumor
LUSC.Normal
MESO.Tumor
OV.Tumor
PAAD.Tumor
PCPG.Tumor
PRAD.Tumor
PRAD.Normal
READ.Tumor
READ.Normal
SARC.Tumor
SKCM.Tumor
SKCM.Metastasis
STAD.Tumor
STAD.Normal
TGCT.Tumor
THCA.Tumor
THCA.Normal
THYM.Tumor
UCEC.Tumor
UCEC.Normal
UCS.Tumor
UVM.Tumor
f
20nM Rapamycin
20nM Rapamycin+
1uM iMDK
HT1376
RT112
SW780
HT1376
Fold Change
days on treatment
DMSO
iMDK
Rapamycin
iMDK+Rapamycin

Supplementary Fig.6 *In vitro* experiment showing SLS cells are rapamycin tolerant, related to Fig. 3.

a. Expression of genes identified as upregulated in AML cells in this study before and after rapamycin treatment in the primary culture.

b. Expression of genes upregulated in SLS (refer to Supplementary Fig. 4f) in the control group.

c. Expression of dormancy marker genes in the control group.

d. Tumor volume relative to pre-treatment tumor volume. Data are presented as mean ± SD. Tumor volume was measure immediately before each treatment. TTJ xenograft mice (n=6 per group) were treated 3 times/wk with DMSO, iMDK (9mg/kg), rapamycin (3mg/kg), or combined iMDK (9mg/kg) and rapamycin (3mg/kg).

e. Expression of MDK across cancer types and matched normal tissues (data obtained from TCGA). Box plots show the first quartile, median, and the third quartile. P-value Significant Codes: 0 ≤ *** < 0.001 ≤ ** < 0.01 ≤ * < 0.05 ≤ . < 0.1. Refer number of RNA-seq datasets for each cancer type: https://www.cancer.gov/about-nci/organization/ccg/research/structural-genomics/tcga/studied-cancers.

f. Cell growth inhibited by combination treatment of rapamycin and iMDK on 3 bladder cancer cell lines. Left panel: microscopy pictures showing growth of 3 bladder cancer cell lines treated with 20nM rapamycin alone (first column) or combination treatment of 20nM rapamycin and 1μM iMKD (second column) for 6 days. Scale bar, 100 μm (for all images). Right panel: cell proliferation, assessed by crystal violet assay, of bladder cell line HT1376 on the treatment of DMSO (vehicle), 20nM rapamycin, 1μM iMDK or combination of 20nM rapamycin and 1μM iMDK for 14 days. All experiments were replicated 3 times. Data are presented as mean ± SD.

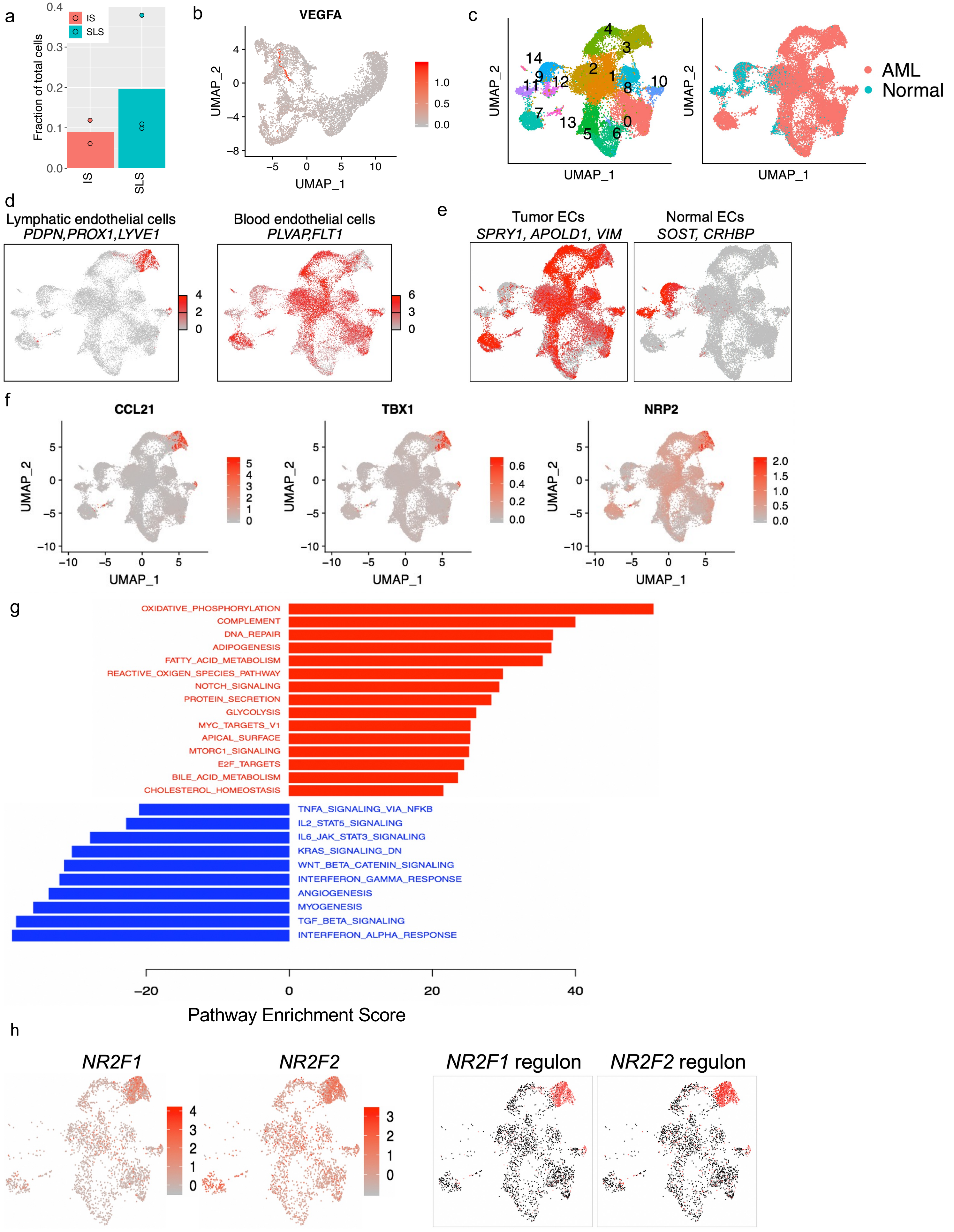
a
IS
SLS
Fraction of total cells
b
VEGFA
UMAP_2
UMAP_1
c
AML
Normal
d
Lymphatic endothelial cells
PDPN,PROX1,LYVE1
Blood endothelial cells
PLVAP,FLT1
e
Tumor ECs
SPRY1, APOLD1, VIM
Normal ECs
SOST, CRHBP
f
CCL21
TBX1
NRP2
g
OXIDATIVE_PHOSPHORYLATION
COMPLEMENT
DNA_REPAIR
ADIPOGENESIS
FATTY_ACID_METABOLISM
REACTIVE_OXIGEN_SPECIES_PATHWAY
NOTCH_SIGNALING
PROTEIN_SECRETION
GLYCOLYSIS
MYC_TARGETS_V1
APICAL_SURFACE
MTORC1_SIGNALING
E2F_TARGETS
BILE_ACID_METABOLISM
CHOLESTEROL_HOMEOSTASIS
TNFA_SIGNALING_VIA_NFKB
IL2_STAT5_SIGNALING
IL6_JAK_STAT3_SIGNALING
KRAS_SIGNALING_DN
WNT_BETA_CATENIN_SIGNALING
INTERFERON_GAMMA_RESPONSE
ANGIOGENESIS
MYOGENESIS
TGF_BETA_SIGNALING
INTERFERON_ALPHA_RESPONSE
Pathway Enrichment Score
h
NR2F1
NR2F2
NR2F1 regulon
NR2F2 regulon

Supplementary Fig.7 Remodeling of endothelial cells, related to Fig. 4.

a. Fraction of endothelial cells in IS (n=2) and SLS (n=3) tumors identified in IHC data.
b. Expression of *VEGFA* in AML cells.
c. Re-clustering of 20,772 endothelial cells (pooled from normal kidney and AML tumor) revealed 14 clusters.
d. Expression of lymphatic endothelial marker genes (left) and blood endothelial marker genes (right) in endothelial cell clusters. One cluster of 646 cells (cluster 3; *PDPN*+, *PROX1*+ and *LYVE1*+) was composed of lymphatic endothelial cells (LECs) and was derived solely from tumor. Fourteen clusters of blood endothelial cells (*FLT1*+ and *PLVAP*+) were identified. Subtypes of endothelial cells were annotated based on published cell markers[119].
e. UMAP showing averaged expression of representative genes specifically expressed in endothelial cells (ECs) derived from AML tumors (left) or from matched normal tissues (right). Eleven of clusters were primarily tumor-derived (*PLVAP*+ and *APOLD1*+) and three clusters were primarily normal kidney-derived (*SOST*+ and *CRHBP*+).
f. Feature plots showing expression of *CCL21* (C-C Motif Chemokine Ligand 21), *TBX1* and *NRP2*. These genes were observed specifically in tumor LECs. High expression of *CCL21* promotes immune cells migration and tumor metastasis[120]. High expression of *TBX1* is required for lymphatic vessel development[121]. NRP2 has been reported to be involved in sprouting lymphangiogenesis[122].
g. Hallmark pathways enriched in tumor-associated endothelial cells. Analysis of hallmark pathway gene signatures comparing all tumor-derived endothelial cells versus normal kidney-derived endothelial cells revealed stronger fatty acid metabolism in tumor-associated endothelial cells. Fatty acid metabolism plays a critical role in lymphatic differentiation and development by providing energy resources for cell proliferation and epigenetic regulation[123].
h. Expression and regulon activity of *NR2F1* and *NR2F2*. Left two panels: expression of *NR2F1* and *NR2F2;* right two panels: regulon activity of these transcription factors. Regulon analysis identified candidates that may underlie these gene expression differences in LECs, including transcription factors such as *NR2F1* and *NR2F2* that are highly expressed in tumor-derived LECs specifically. The genetic networks regulated by these transcription factors also showed high activity in LECs. NR2F2 is of particular interest in LAM because of its identification as a GWAS susceptibility locus[124]. NR2F2 has been shown to physically and functionally interact with PROX1 to regulate LEC-fate specification[125,126] and promote the formation of lymphatic vasculature[122]. NR2F2 controls sprouting lymphangiogenesis by transcriptional up-regulation of NRP2, a co-receptor for VEGF-C[127]. NR2F1 has recently been shown to be required for vascular development[128].

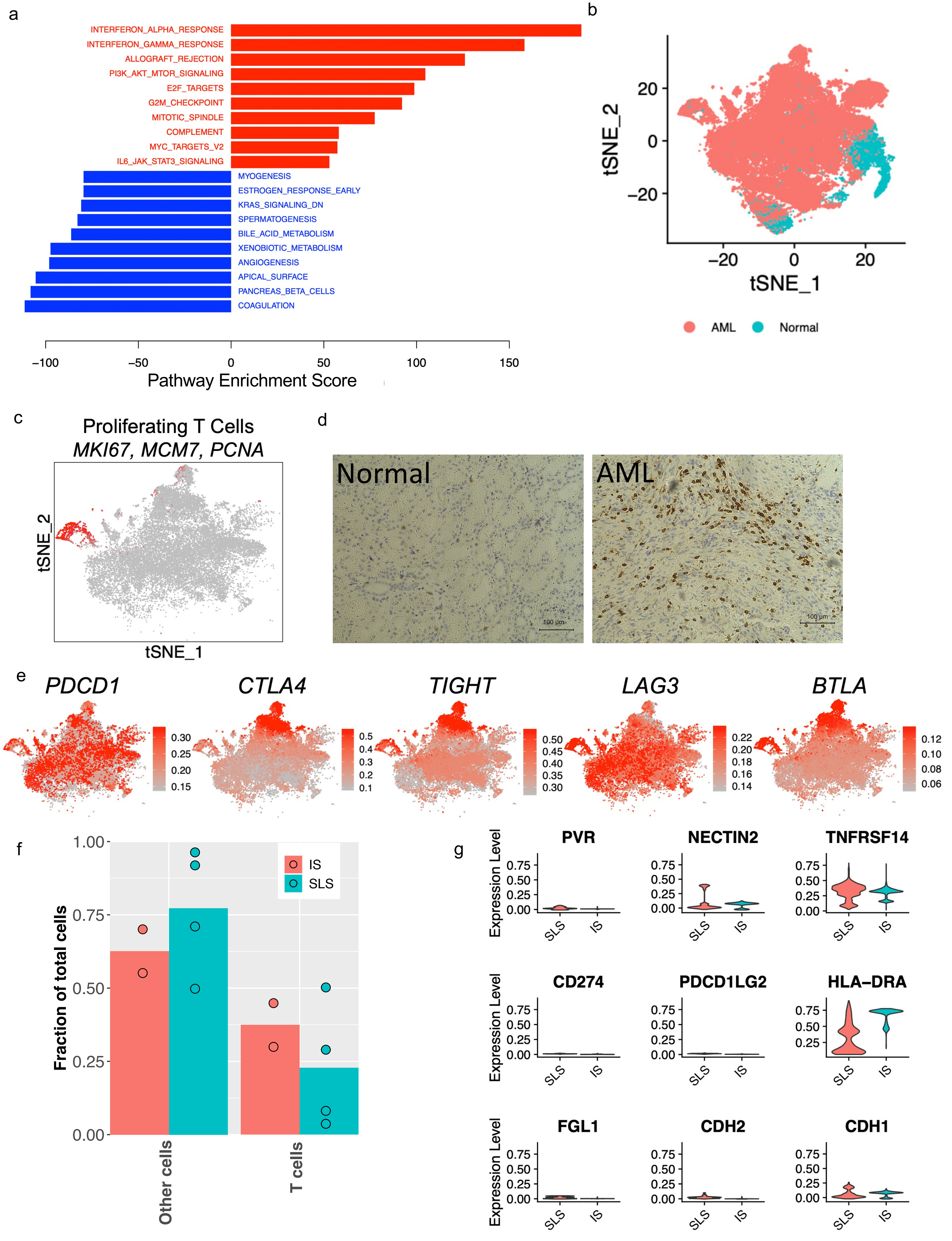
a
INTERFERON_ALPHA_RESPONSE
INTERFERON_GAMMA_RESPONSE
ALLOGRAFT_REJECTION
PI3K_AKT_MTOR_SIGNALING
E2F_TARGETS
G2M_CHECKPOINT
MITOTIC_SPINDLE
COMPLEMENT
MYC_TARGETS_V2
IL6_JAK_STAT3_SIGNALING
MYOGENESIS
ESTROGEN_RESPONSE_EARLY
KRAS_SIGNALING_DN
SPERMATOGENESIS
BILE_ACID_METABOLISM
XENOBIOTIC_METABOLISM
ANGIOGENESIS
APICAL_SURFACE
PANCREAS_BETA_CELLS
COAGULATION
−100 −50 0 50 100 150
Pathway Enrichment Score
b
tSNE_2
tSNE_1
AML
Normal
c
Proliferating T Cells
MKI67, MCM7, PCNA
tSNE_2
tSNE_1
d
Normal
AML
e
PDCD1
CTLA4
TIGHT
LAG3
BTLA
f
Fraction of total cells
IS
SLS
Other cells
T cells
g
Expression Level
PVR
NECTIN2
TNFRSF14
CD274
PDCD1LG2
HLA−DRA
FGL1
CDH2
CDH1
SLS
IS

Supplementary Fig.8 Characterization of T cells related to Fig. 5.

a. Pathways enriched in T cells from tumor samples compared to those from matched normal tissues. Red bars represent pathways enriched in T cells from tumor samples; blue bars represent pathways enriched in T cells from matched normal tissues.
b. UMAP plot of cell origin.
c. Averaged expression of *MKI67*, *MCM7*, and *PCNA* in T cells.
d. Representative images of CD3 IHC of 5 AML tumors and 4 matched normal samples.
e. Feature plots showing expression of checkpoint markers in T cells.
f. Bar plot showing fraction of T cells in SLS (n=4) versus IS (n=2) dominant tumors.
g. Expression profiles of immune checkpoint ligands: TIGIT ligands (*PVR*, *NECTIN2*), BTLA ligand (*TNFRSF14*), LAG3 ligand (*HLA-DRA*, *FGL1*), *KLRG1* ligand (*CDH1*, *CDH2*), and PD-1 ligands (*CD274*, *PDCD1LG2*). The y axis represents the normalized gene expression value.

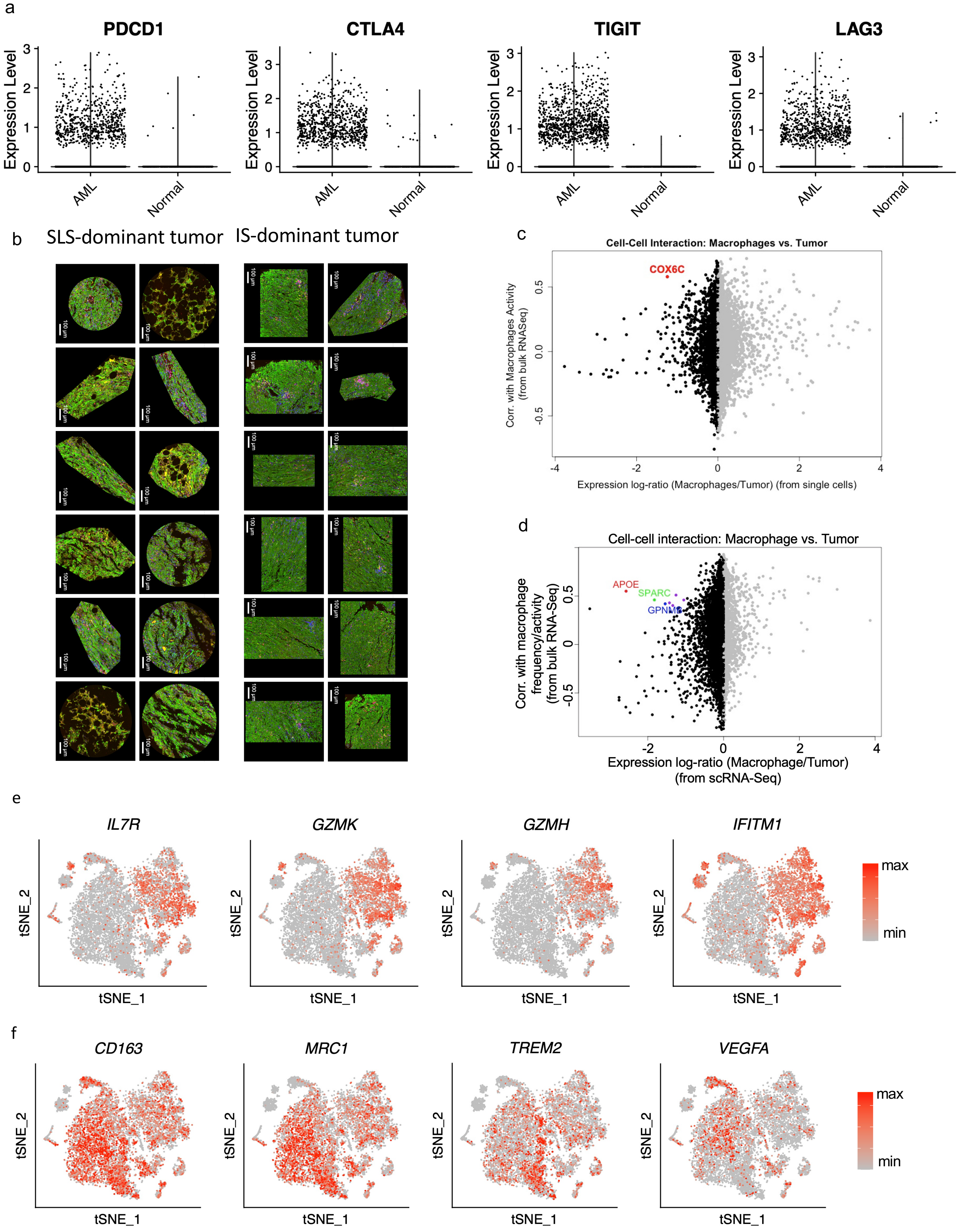
a
PDCD1
CTLA4
TIGIT
LAG3
Expression Level
AML
Normal
b
SLS-dominant tumor
IS-dominant tumor
c
Cell-Cell Interaction: Macrophages vs. Tumor
COX6C
Corr. with Macrophages Activity (from bulk RNASeq)
Expression log-ratio (Macrophages/Tumor) (from single cells)
d
Cell-cell interaction: Macrophage vs. Tumor
APOE
SPARC
GPNMB
Corr. with macrophage frequency/activity (from bulk RNA-Seq)
Expression log-ratio (Macrophage/Tumor) (from scRNA-Seq)
e
IL7R
GZMK
GZMH
IFITM1
tSNE_1
tSNE_2
max
min
f
CD163
MRC1
TREM2
VEGFA
tSNE_1
tSNE_2
max
min

Supplementary Fig.9 Suppressive immune microenvironment in AML, related to Fig. 6.

a. Violin plot of expression of immune checkpoint genes in macrophages obtained from tumors or from matched normal kidneys.
b. Representative images of Nanostring 12 ROIs of one SLS-dominant tumor and 12 ROIs of one IS-dominant tumor. Scale bars, 100 μm.
c. Inferred interactions between tumor cells and macrophages calculated by integrative analysis of spatial transcriptomics of the representative IS-dominant tumor (12 ROIs) and scRNA-Seq. x-axis displays relative expression of genes in single cell data. Only genes that are expressed in both single cell data and spatial transcriptomics data are shown. Left side are genes relatively highly expressed in tumor cells; right side are genes relatively highly expressed in macrophages. Y-axis displays Pearson Correlation Coefficient (PCC) of gene expression with macrophage frequency in spatial transcriptomics data. Genes with log-ratio less than -1.5 and correlation coefficient higher than 0.4 are colored. *APOE*: PCC=0.49, p=0.1 (correlation test, two-sided).
d. Similar analysis as in Fig. 6d, but calculated by integrative analysis of bulk RNA-seq data from a published dataset[8] and scRNA-seq. *APOE*: PCC=0.55, p=0.1 (correlation test, two-sided).
e. Feature plots showing expression of *IL7R*, *GZMK*, *GZMH*, *IFITM1* in macrophages.
f. Feature plots showing expression of marker genes of tumor-associated macrophages, including *CD163*, *MRC1*, *TREM2*, *VEGFA*.

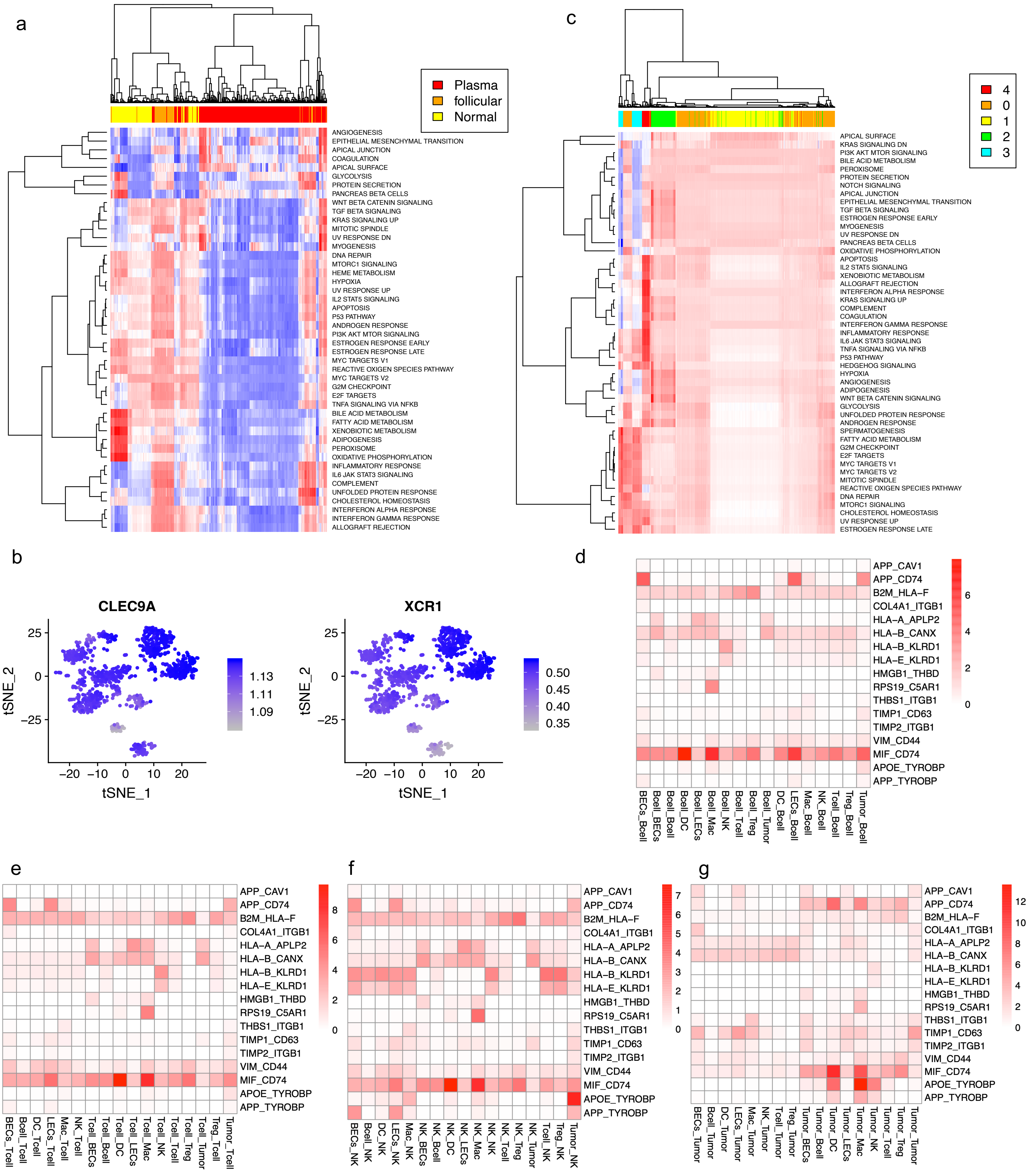
a
Plasma
follicular
Normal
ANGIOGENESIS
EPITHELIAL MESENCHYMAL TRANSITION
APICAL JUNCTION
COAGULATION
APICAL SURFACE
GLYCOLYSIS
PROTEIN SECRETION
PANCREAS BETA CELLS
WNT BETA CATENIN SIGNALING
TGF BETA SIGNALING
KRAS SIGNALING UP
MITOTIC SPINDLE
UV RESPONSE DN
MYOGENESIS
DNA REPAIR
MTORC1 SIGNALING
HEME METABOLISM
HYPOXIA
UV RESPONSE UP
IL2 STAT5 SIGNALING
APOPTOSIS
P53 PATHWAY
ANDROGEN RESPONSE
PI3K AKT MTOR SIGNALING
ESTROGEN RESPONSE EARLY
ESTROGEN RESPONSE LATE
MYC TARGETS V1
REACTIVE OXIGEN SPECIES PATHWAY
MYC TARGETS V2
G2M CHECKPOINT
E2F TARGETS
TNFA SIGNALING VIA NFKB
BILE ACID METABOLISM
FATTY ACID METABOLISM
XENOBIOTIC METABOLISM
ADIPOGENESIS
PEROXISOME
OXIDATIVE PHOSPHORYLATION
INFLAMMATORY RESPONSE
IL6 JAK STAT3 SIGNALING
COMPLEMENT
UNFOLDED PROTEIN RESPONSE
CHOLESTEROL HOMEOSTASIS
INTERFERON ALPHA RESPONSE
INTERFERON GAMMA RESPONSE
ALLOGRAFT REJECTION
c
4
0
1
2
3
APICAL SURFACE
KRAS SIGNALING DN
PI3K AKT MTOR SIGNALING
BILE ACID METABOLISM
PEROXISOME
PROTEIN SECRETION
NOTCH SIGNALING
APICAL JUNCTION
EPITHELIAL MESENCHYMAL TRANSITION
TGF BETA SIGNALING
ESTROGEN RESPONSE EARLY
MYOGENESIS
UV RESPONSE DN
PANCREAS BETA CELLS
OXIDATIVE PHOSPHORYLATION
APOPTOSIS
IL2 STAT5 SIGNALING
XENOBIOTIC METABOLISM
ALLOGRAFT REJECTION
INTERFERON ALPHA RESPONSE
KRAS SIGNALING UP
COMPLEMENT
COAGULATION
INTERFERON GAMMA RESPONSE
INFLAMMATORY RESPONSE
IL6 JAK STAT3 SIGNALING
TNFA SIGNALING VIA NFKB
P53 PATHWAY
HEDGEHOG SIGNALING
HYPOXIA
ANGIOGENESIS
ADIPOGENESIS
WNT BETA CATENIN SIGNALING
GLYCOLYSIS
UNFOLDED PROTEIN RESPONSE
ANDROGEN RESPONSE
SPERMATOGENESIS
FATTY ACID METABOLISM
G2M CHECKPOINT
E2F TARGETS
MYC TARGETS V1
MYC TARGETS V2
MITOTIC SPINDLE
REACTIVE OXIGEN SPECIES PATHWAY
DNA REPAIR
MTORC1 SIGNALING
CHOLESTEROL HOMEOSTASIS
UV RESPONSE UP
ESTROGEN RESPONSE LATE
b
CLEC9A
25
0
−25
−20 −10 0 10 20
tSNE_1
tSNE_2
1.13
1.11
1.09
XCR1
0.50
0.45
0.40
0.35
d
APP_CAV1
APP_CD74
B2M_HLA−F
COL4A1_ITGB1
HLA−A_APLP2
HLA−B_CANX
HLA−B_KLRD1
HLA−E_KLRD1
HMGB1_THBD
RPS19_C5AR1
THBS1_ITGB1
TIMP1_CD63
TIMP2_ITGB1
VIM_CD44
MIF_CD74
APOE_TYROBP
APP_TYROBP
6
4
2
0
BECs_Bcell
Bcell_BECs
Bcell_Bcell
Bcell_DC
Bcell_LECs
Bcell_Mac
Bcell_NK
Bcell_Tcell
Bcell_Treg
Bcell_Tumor
DC_Bcell
LECs_Bcell
Mac_Bcell
NK_Bcell
Tcell_Bcell
Treg_Bcell
Tumor_Bcell
e
8
6
4
2
0
BECs_Tcell
Bcell_Tcell
DC_Tcell
LECs_Tcell
Mac_Tcell
NK_Tcell
Tcell_BECs
Tcell_Bcell
Tcell_DC
Tcell_LECs
Tcell_Mac
Tcell_NK
Tcell_Tcell
Tcell_Treg
Tcell_Tumor
Treg_Tcell
Tumor_Tcell
f
7
6
5
4
3
2
1
0
BECs_NK
Bcell_NK
DC_NK
LECs_NK
Mac_NK
NK_BECs
NK_Bcell
NK_DC
NK_LECs
NK_Mac
NK_NK
NK_Tcell
NK_Treg
NK_Tumor
Tcell_NK
Treg_NK
Tumor_NK
g
12
10
8
6
4
2
0
BECs_Tumor
Bcell_Tumor
DC_Tumor
LECs_Tumor
Mac_Tumor
NK_Tumor
Tcell_Tumor
Treg_Tumor
Tumor_BECs
Tumor_Bcell
Tumor_DC
Tumor_LECs
Tumor_Mac
Tumor_NK
Tumor_Tcell
Tumor_Treg
Tumor_Tumor

Supplementary Fig.10 Molecular interactions between tumor and tumor microenvironment inferred by ligand-receptor co-expression, related to Fig. 7.

a. Pathways enriched in plasma cells and follicular B cells obtained from tumors, and enriched in B cells obtained from matched normal tissues.
b. Feature plots showing expression of *CLEC9A* and *XCR1* in dendritic cells.
c. Pathways enriched in each cluster of dendritic cells colored by column bar.
d. Interactions between B cells and other cell types in SLS-dominant tumors, calculated as the product of the average ligand expression and average receptor expression. Only interactions with a score greater than 1 across any cell type pair are displayed. Each column shows a pair of cell types, and each row shows the ligand-receptor pair. The color indicates interaction score. Column label: cell type expressing the ligand and cell type expressing the receptor are separated by "_". Row label: ligand and receptor are separated by "_".
e. Interactions between T cells and other cell types in SLS-dominant tumors, calculated as the product of the average ligand expression and average receptor expression. Only interactions with a score greater than 1 across any cell type pair are displayed.
f. Interactions between NK cells and other cell types in SLS-dominant tumors. Only interactions with a score greater than 1 across any cell type pair are displayed.
g. Interactions between tumor cells and other cell types in SLS-dominant tumors. Only interactions with a score greater than 1 across any cell type pair are displayed.

Supplementary table 1. Patient information.

AML ID	mutation
AML1098	TSC1 c.1798C>T p.Q600* at 79%
AML1162	TSC2 c2974 C>T (p.Q992*), exon 26 – in 41.33% of 1196 reads; TSC2 c5226 indels (), exon 40 – in 12.86% of 916 reads
AML1166	TSC2 c.4537G>T (p.E1513*), exon 35 - in 35% of 343 reads; TSC2 c.4442dup (p.S1482Efs*42), exon 34 - in 36% of 297 reads
AML1167	TSC2 c.1372C>T (p.R458*), exon 13 – in 47.56% of 1214 reads
AML1171	TSC2 c.1111_1119+1delinsTC (), exon 11 - in 19% of 146 reads
AML1172	TSC1 c.2074C>T (p.R692*), exon 17 - in 12% of 316 reads; TSC1 c.989_1011dup (p.I338*), exon 10 - in 4% of 304 reads
