# Supplementary material for: Midkine expression by stem-like tumor cells drives persistence to mTOR inhibition and an immune-suppressive microenvironment
Source: Nat Commun. 2022 Aug 26;13:5018. doi: 10.1038/s41467-022-32673-7 (PMC9418323; doi:10.1038/s41467-022-32673-7)
Supplement: Supplementary file 2 — Description of Additional Supplementary Files [file 41467_2022_32673_MOESM2_ESM.pdf]

File Name: Supplementary Data 1
Description: Differentially expressed genes in Tumor, TAF and normal mesenchymal cells. Each of cell type was compared to the other two cell types separately.

File Name: Supplementary Data 2
Description: Enriched regulons in SLS tumor cells compared to matched normal mesenchymal cells.

File Name: Supplementary Data 3
Description: Enriched regulons in IS tumor cells compared to matched normal mesenchymal cells.

File Name: Supplementary Data 4
Description: Marker genes of each cluster of primary culture cells.

File Name: Supplementary Data 5
Description: Re-defined gene signatures for each major cell type based on scRNA-Seq data.

File Name: Supplementary Data 6
Description: Identified genes mediating cell-cell interactions.

File Name: Supplementary Data 7
Description: Molecular interactions identified between TAF and SLS or IS tumor cells.
