# Supplementary material for: Midkine expression by stem-like tumor cells drives persistence to mTOR inhibition and an immune-suppressive microenvironment
Source: Nat Commun. 2022 Aug 26;13:5018. doi: 10.1038/s41467-022-32673-7 (PMC9418323; doi:10.1038/s41467-022-32673-7)
Supplement: Supplementary file 10 — Reporting Summary [file 41467_2022_32673_MOESM10_ESM.pdf]

# nature portfolio

Corresponding author(s): Elizabeth P. Henske

Last updated by author(s): Jun 7, 2022

# Reporting Summary

Nature Portfolio wishes to improve the reproducibility of the work that we publish. This form provides structure for consistency and transparency in reporting. For further information on Nature Portfolio policies, see our Editorial Policies and the Editorial Policy Checklist.

## Statistics

For all statistical analyses, confirm that the following items are present in the figure legend, table legend, main text, or Methods section.

| n/a | Confirmed | |
|---|---|---|
| ☐ | ☒ | The exact sample size (*n*) for each experimental group/condition, given as a discrete number and unit of measurement |
| ☐ | ☒ | A statement on whether measurements were taken from distinct samples or whether the same sample was measured repeatedly |
| ☐ | ☒ | The statistical test(s) used AND whether they are one- or two-sided<br>*Only common tests should be described solely by name; describe more complex techniques in the Methods section.* |
| ☒ | ☐ | A description of all covariates tested |
| ☐ | ☒ | A description of any assumptions or corrections, such as tests of normality and adjustment for multiple comparisons |
| ☐ | ☒ | A full description of the statistical parameters including central tendency (e.g. means) or other basic estimates (e.g. regression coefficient) AND variation (e.g. standard deviation) or associated estimates of uncertainty (e.g. confidence intervals) |
| ☐ | ☒ | For null hypothesis testing, the test statistic (e.g. *F*, *t*, *r*) with confidence intervals, effect sizes, degrees of freedom and *P* value noted<br>*Give P values as exact values whenever suitable.* |
| ☒ | ☐ | For Bayesian analysis, information on the choice of priors and Markov chain Monte Carlo settings |
| ☒ | ☐ | For hierarchical and complex designs, identification of the appropriate level for tests and full reporting of outcomes |
| ☐ | ☒ | Estimates of effect sizes (e.g. Cohen's *d*, Pearson's *r*), indicating how they were calculated |

*Our web collection on statistics for biologists contains articles on many of the points above.*

## Software and code

Policy information about availability of computer code

| | |
|---|---|
| Data collection | R (version 4.0.2) were included for data collection in this research. |
| Data analysis | GraphPad Prism (version 9.2.0.283), R package Seurat (version 4.0.2), CellRanger (version 3.0.2), GSVA (version 1.36.3), and SCENIC (version 1.2.2) were included for data analysis in this research. |

For manuscripts utilizing custom algorithms or software that are central to the research but not yet described in published literature, software must be made available to editors and reviewers. We strongly encourage code deposition in a community repository (e.g. GitHub). See the Nature Portfolio guidelines for submitting code & software for further information.

## Data

Policy information about availability of data

All manuscripts must include a data availability statement. This statement should provide the following information, where applicable:

- Accession codes, unique identifiers, or web links for publicly available datasets
- A description of any restrictions on data availability
- For clinical datasets or third party data, please ensure that the statement adheres to our policy

The raw scRNA-seq data of 5 LAM samples, 6 AML and 4 matched normal kidney samples generated in this study are available in GEO (GSE190260). The raw scTCR-seq data of 4 tumors and 10X Genomics spatial transcriptomics data generated in this study are available in GEO (GSE208262). The raw Nanostring whole transcriptome digital spatial profiling data generated in this study are available in GEO (GSE210755). The publicly available bulk RNA-seq data from TSC patients8 used in this study are available in the Database of Genotypes and Phenotypes (dbGaP) under the accession code phs001357.v1.p1 (https://www.ncbi.nlm.nih.gov/projects/gap/cgi-bin/study.cgi?study_id=phs001357.v1.p1).
GRCh38 reference genome: https://support.10xgenomics.com/single-cell-gene-expression/software/downloads/latest. Source data are provided with this paper.

The remaining data are available within the Article, Supplementary Information or Source Data file.

# Field-specific reporting

Please select the one below that is the best fit for your research. If you are not sure, read the appropriate sections before making your selection.

- [x] Life sciences
- [ ] Behavioural & social sciences
- [ ] Ecological, evolutionary & environmental sciences

For a reference copy of the document with all sections, see nature.com/documents/nr-reporting-summary-flat.pdf

# Life sciences study design

All studies must disclose on these points even when the disclosure is negative.

| | |
|---|---|
| Sample size | For single cell analysis, sample size was determined by the availability of patient samples. For in vitro studies, we used empirical n=3 replicates. Based on large effect size we observed in in vitro studies, we determined that for in vivo study, n=6 can achieve a statistic power of 0.8 to detect difference between groups using two-sided t-test at significance of 0.05 at any time points. |
| Data exclusions | For single cell analysis, cells were filtered from downstream analysis with the criteria of < 200 genes or > 6000 genes detected and > 0.1 fraction of mitochondrial gene. |
| Replication | All experiments in this study were repeated at least 3 times. All replicates showed similar results. |
| Randomization | All mice and cell culture experiments were randomly assigned to treatment groups. |
| Blinding | Blinding was performed during data collection and data analysis for single cell experiments Quantification of IHC and IF was performed blindly. |

# Reporting for specific materials, systems and methods

We require information from authors about some types of materials, experimental systems and methods used in many studies. Here, indicate whether each material, system or method listed is relevant to your study. If you are not sure if a list item applies to your research, read the appropriate section before selecting a response.

## Materials & experimental systems

| n/a | Involved in the study | |
|---|---|---|
| ☐ | ☒ | Antibodies |
| ☐ | ☒ | Eukaryotic cell lines |
| ☒ | ☐ | Palaeontology and archaeology |
| ☐ | ☒ | Animals and other organisms |
| ☐ | ☒ | Human research participants |
| ☒ | ☐ | Clinical data |
| ☒ | ☐ | Dual use research of concern |

## Methods

| n/a | Involved in the study | |
|---|---|---|
| ☒ | ☐ | ChIP-seq |
| ☒ | ☐ | Flow cytometry |
| ☒ | ☐ | MRI-based neuroimaging |

## Antibodies

| | |
|---|---|
| Antibodies used | For multiplex IF:<br>anti-human TAGLN, abcam (ab14106): 1ug/ml<br>anti-human MDK, abcam (ab52637): 1:100<br>anti-human CTSK, boster (PB9856): 0.5ug/ml<br>For IHC:<br>anti-human CD68 (clone PG-M1), Dako (M0876): 1:100<br>anti-human CD3 (clone F7.2.38), Dako (A0452): 1:50<br>anti-human CD31(clone JC70A), Dako (M0823): 1:50<br>For Nanostring IF:<br>anti-human a-SMA (clone 1A4), invitrogen (53-9760-82), 1:400<br>anti-human CD68(clone KP1) , Santa Cruz (sc-20060AF594), 1:400<br>anti-human CD3e (clone UMAB54), Origene (UM500048): 1:200 |
| Validation | anti-human TAGLN, abcam (ab14106): https://www.abcam.com/TAGLNTransgelin-antibody-ab14106.html?gclsrc=aw.ds%7Caw.ds&gclid=CjwKCAjw7vuUBhBUEiwAEdu2pOnBfZ3n9lhjl0L1f7nPGLTz7s3THvhBT8mdRU6gt3Ic9SKLkQD9BxoC0d0QAvD_BwE. The antibody was validated by the manufacturer for western blotting, immunohistochemistry and immunofluorescence. Positive control: WB: Human primary smooth muscle cells; HeLa whole cell lysate; Mouse colon tissue lysate; Rat colon tissue lysate. ICC: HeLa cells; mouse muscle cells.<br>anti-human MDK, abcam (ab52637): https://www.abcam.com/midkine-antibody-ep1143y-ab52637.html. The antibody was validated by the manufacturer for western blotting, immunohistochemistry, immunofluorescence, immunoprecipitation, and flow cytometry. |

Positive control: Human liver carcinoma; Human pancreatic carcinoma; HeLa cells; SH-SY5Y (Human neuroblastoma cell line from bone marrow).
anti-human CTSK, boster (PB9856): https://www.bosterbio.com/anti-cathepsin-k-picoband-trade-antibody-pb9856-boster.html. The antibody was validated by the manufacturer for western blotting, immunohistochemistry, immunofluorescence, and ELISA with known positive and negative samples to ensure specificity and high affinity.
anti-human CD68 (clone PG-M1), Dako (M0876): https://www.citeab.com/antibodies/2414880-m0876-cd68-concentrate. The antibody was validated by the manufacturer for immunohistochemistry, immunofluorescence, flow cytometry. 535 citations.
anti-human CD3 (clone F7.2.38), Dako (A0452): https://www.citeab.com/antibodies/3382891-a0452-cd3. The antibody was validated by the manufacturer for immunohistochemistry, immunofluorescence, flow cytometry. 888 citations.
anti-human CD31(clone JC70A), Dako (M0823): https://www.citeab.com/antibodies/2414851-m0823-cd31-endothelial-cell-concentrate. The antibody was validated by the manufacturer for immunohistochemistry, immunofluorescence, flow cytometry. 642 citations.
anti-human a-SMA, invitrogen (53-9760-82): https://www.thermofisher.com/antibody/product/Alpha-Smooth-Muscle-Actin-Antibody-clone-1A4-Monoclonal/53-9760-82. The antibody was validated by the manufacturer for immunohistochemistry, immunofluorescence, flow cytometry.

# Eukaryotic cell lines

Policy information about cell lines

Cell line source(s)

Patient-derived TSC2-deficient cell line 621-101, TSC2-addback cell line 621-103, mouse kidney derived TSC2-null cell line TTJ (the gift of Vera Krymskaya) and TSC2-add back cell line TTJ-TSC2 are maintained in our lab. The normal human lung fibroblasts NHLF (CC-2512) was purchased from Lonza Group (Switzerland).

Authentication

TSC2-null and TSC2-add back cell lines are routinely authenticated using western immunoblotting and qPCR to confirm TSC2 protein loss and expression before any experiment.

Mycoplasma contamination

All cells used in this study were tested negative for mycoplasma contamination. All cells in our laboratory are monthly tested for mycoplasma contamination.

Commonly misidentified lines
(See ICLAC register)

No commonly misidentified lines were used in this study.

# Animals and other organisms

Policy information about studies involving animals; ARRIVE guidelines recommended for reporting animal research

Laboratory animals

Seven-week-old female athymic nude mice (Crl:NU(NCr)-Foxn1nu) were obtained from Charles River Laboratory.

Wild animals

No wild animals were used in the study.

Field-collected samples

No field-collected samples were used in the study.

Ethics oversight

Animal studies were approved by the Brigham and Women's Hospital Animal Care and Use Committee (IACUC). All husbandry and experiment procedures with mice were conducted in accordance with protocols approved.

Note that full information on the approval of the study protocol must also be provided in the manuscript.

# Human research participants

Policy information about studies involving human research participants

Population characteristics

Six AML and five LAM samples were collected for this study. Six AML patients (5 female and 1 male) age from 32 to 51. Five LAM female patients age from 52 to 72. None of these patients received rapamycin treatment within 6 months before surgery.

Recruitment

LAM specimens, AML tumor samples and matched normal kidneys were collected by a clinical coordinator at Brigham and Women's Hospital. All patients provided informed consent.

Ethics oversight

IRB approval from Brigham and Women's Hospital.

Note that full information on the approval of the study protocol must also be provided in the manuscript.
